# Analysis of the characteristics of chemotherapy-resistant renal cell carcinomas based on global transcriptional analysis of their tissues and cell lines

**DOI:** 10.1371/journal.pone.0225721

**Published:** 2019-11-27

**Authors:** Takahiro Isono, Masafumi Suzaki

**Affiliations:** Central Research Laboratory, Shiga University of Medical Science, Otsu, Shiga, Japan; Uppsala University, SWEDEN

## Abstract

Starvation-resistant renal cell carcinoma (RCC) cell lines are considered dormant-state cells that survive even under glucose starvation. The cellular biological and global transcriptional analysis using these cells identified potential markers of chemotherapy-resistant RCC and therapeutic agent candidates. Recently, we showed that ARL4C was a predictive biomarker for poor prognosis in patients with chemotherapy-resistant RCC by the global transcriptional analysis of patient primary tissues. The objective of this study was to identify the characteristics of chemotherapy-resistant RCC by the global transcriptional analysis of primary tissues of patients with RCC and RCC cell lines. The connective global transcriptional analysis showed that two starvation-resistant RCC cell lines, SW839 and KMRC-1, were strongly correlated to tissues of patients with chemotherapy-resistant RCC and showed high expressions of invasive- and proliferation-related genes. We found fibronectin (*FN1)* expression was a predictive biomarker in some patients with chemotherapy-resistant RCC, which especially correlated with two starvation-resistant RCC cell lines. These results indicate these cell lines emulate chemotherapy-resistant RCC and might be useful in the search for markers to predict poor prognosis and in the development of therapeutic agents and their index markers for chemotherapy-resistant RCCs.

## Introduction

Renal cell carcinoma (RCC) is the most common renal malignancy and its incidence is currently increasing [1]. More than 30% of newly diagnosed cases are regionally-advanced or at metastatic stages. Radical nephrectomy remains the standard and only curative treatment for patients with localized RCC. However, up to half of nephrectomized patients that appear cured eventually develop distant metastases [2]. Therefore, effective anticancer drugs for metastatic RCC have been investigated, and several new molecular targeting drugs, including tyrosine kinase and mTOR inhibitors, have been developed [3–9]. However, the therapeutic efficiencies of these agents are insufficient.

Previously, we demonstrated the presence of two types of cells in RCC involved in carbon metabolism and cell signaling under glucose starvation, which is the major nutrient denied to cells following the inhibition of angiogenesis [10]. These findings suggested that differences between starvation-resistant and starvation-sensitive RCC cells might be key factors in developing novel targeted therapies. Starvation-resistant cells are dormant-state cells that survive even under glucose starvation [10]. Cell biological analysis and global transcriptional analysis using these two types of RCC cells indicated that mitochondrial manganese-dependent superoxide dismutase (SOD2) [11] and tumor necrosis factor (TNF)-related apoptosis-induced ligand (*TNFSF10/*TRAIL) [12] were potential markers of poor prognosis. In addition, buformin (a biguanide) [10, 11], etomoxir (an inhibitor of beta-oxidation from fatty acids) [11], and chetomin (a nuclear inhibitor of hypoxia inducible factor [HIF]) [13], may be potential therapeutic agents for RCC. Recently, we showed that seven genes searched for by the global transcriptional analysis of primary tissues from patients with RCC were useful to predict a poor prognosis in patients with chemotherapy-resistant RCC. Furthermore, *ARL4C*, one of these seven genes, was demonstrated to be a useful predictor of a poor prognosis in patients with chemotherapy-resistant RCC by global transcriptomic analyses. Cases with high *ARL4C* expression were associated with a significantly shorter survival periods than in the cases with low *ARL4C* expression (log-rank test, p < 0.001; 8.7 months vs not reached, respectively), and Cox univariate and multivariate analyses showed that high *ARL4C* expression accurately predicted poor survival in this cohort (hazard ratio = 111 and 167, p < 0.001 and p = 0.005, respectively). Moreover, these finding were independently confirmed by clinicopathological analyses of another clinical cohort [14]. However, RCC patients with high *ARL4C* expression are resistant to treatment with tyrosine kinase and mTOR inhibitors. Therefore, novel therapeutic agents targeting other molecules will be required for the treatment of RCC patients.

In this study, we identified the characteristics of chemotherapy-resistant RCCs by the global transcriptional analysis of primary tissues from patients with RCC and RCC cell lines. We showed that starvation-resistant cells might be useful for the development of predictive biomarkers for a poor prognosis, the development of therapeutic agents, and index markers in patients with chemotherapy-resistant RCC.

## Materials and methods

### Cell lines and cell culture conditions

Three starvation-resistant RCC cell lines (SW839, VMRC-RCW and KMRC-1) and four starvation-sensitive RCC cell lines (Caki1, Caki2, NC65 and ACHN) were used in this study. These cell lines were purchased from either the American Type Culture Collection, Riken Cell Bank, Cell Resource Center for Biomedical Research in Tohoku University (Sendai, Japan) or the Japanese Collection of Research Bioresources (Japan). All cell lines were maintained in RPMI 1640 (Nacalai Tesque, Kyoto, Japan), containing 25 mM glucose, supplemented with 10% fetal calf serum, penicillin (100 U/ml) and streptomycin (100 μg/ml) at 37°C in a humidified 5% CO_2_ atmosphere.

### RNA preparation

Total RNA was extracted from seven RCC cell lines using the Trizol Plus RNA Purification kit (Thermo Fisher Scientific, Waltham, MA, USA). Total RNA was quantified using a Bioanalyzer (Agilent, Santa Clara, CA, USA) according to the manufacturer’s instructions. The RNA Integrity Numbers of all prepared total RNA samples were over 8.0.

### High-throughput DNA sequencing

A library of template molecules for high-throughput DNA sequencing was converted from total RNA using the TruSeq RNA Sample Prep Kitv2 (Illumina, San Diego, CA, USA) according to the manufacturer’s protocol. The library was quantified using a Bioanalyzer (Agilent) following the manufacturer’s instructions. The library (4 pM) was subjected to cluster generation on a Single Read Flow Cell v4 (TruSeq SR Cluster Kit v2-cBot-GA) with a cBot generation instrument (Illumina). Sequencing was performed on a Genome Analyzer GAIIx for 58 cycles using Cycle Sequencing v5-GA reagents (Illumina).

### Data analysis

Image analysis and base calling were performed using Real Time Analysis version 1.13 (Illumina). The sequence libraries for each sample were processed using CASAVA Software 1.8.2 (Illumina) to produce 51-bp sequence data in fastq format. The fastq files were processed using Cutadapt version 1.2.1 [15] with option–q 30. In addition, we removed reads shorter than 49-bp using Cutadapt. Trimmed reads for each sample were aligned to the reference genome (Ensembl build GRCh37) using TopHat version 2.0.10 [16] with the default setting, except for option–G. Differential gene expression analysis was performed using Cufflinks [17] with option -g. Cuffmerge was used to merge the assembled transcripts into a consensus gene track from all mapped samples with options–s and–g. Moreover, Cuffqunt was used for quantification using option–M. Cuffdiff was used to identify differentially expressed genes and transcripts between two groups containing 43 primary tissues of patients with RCC and seven RCC cell lines. Genes and transcripts were identified as being significantly differentially and are expressed with q values <0.05, calculated using the Benjamin–Hochberg FDR correction [17]. In addition, the values of fragments per kilobase of exon per million fragments mapped (FPKM) were converted from count values for the comparison of expression levels among genes. The cor_plot and prcomp package of free software R (https://www.r-project.org/) using genes.count_table following Cuffnorm were used to identify the correlation rates and the principal component analysis of 50 samples containing 43 primary tissues of patients with RCC and seven RCC cell lines, respectively. Kyoto Encyclopedia of Genes and Genomes (KEGG) pathway analysis and gene ontology (GO) analysis were performed using DAVID Bioinformatics Resources 6.8 [18]. The data of 43 primary tissues from patients with RCC were obtained from previous data deposited in the DDBJ Japanese Genotype-phenotype Archive for genetic and phenotypic human data under accession number JGAS00000000149. Data from seven RCC cell lines were deposited in the DDBJ under accession number DRA008476.

### The box and whisper plot

Box and whisper plots were generated using a function of boxplot in free software R.

### Receiver operating characteristic (ROC) analysis

ROC curve analysis was performed using the ROCR package in free software R and the maximum accuracy was calculated.

### Statistical analysis

Data were reported as the mean ± standard error (SE). The values were derived from at least three experiments. Statistical analyses were performed using R. One-way factorial analysis of variance (ANOVA) accompanied by pair-wise comparisons using *t*-tests with a pooled standard deviation (SD) was used to compare the means of multiple groups. A p value < 0.05 denotes statistical significance.

## Results

### The correlation rates of global transcriptomic expression in primary tissues of patients with RCC and RCC cell lines

**Fig 1:**
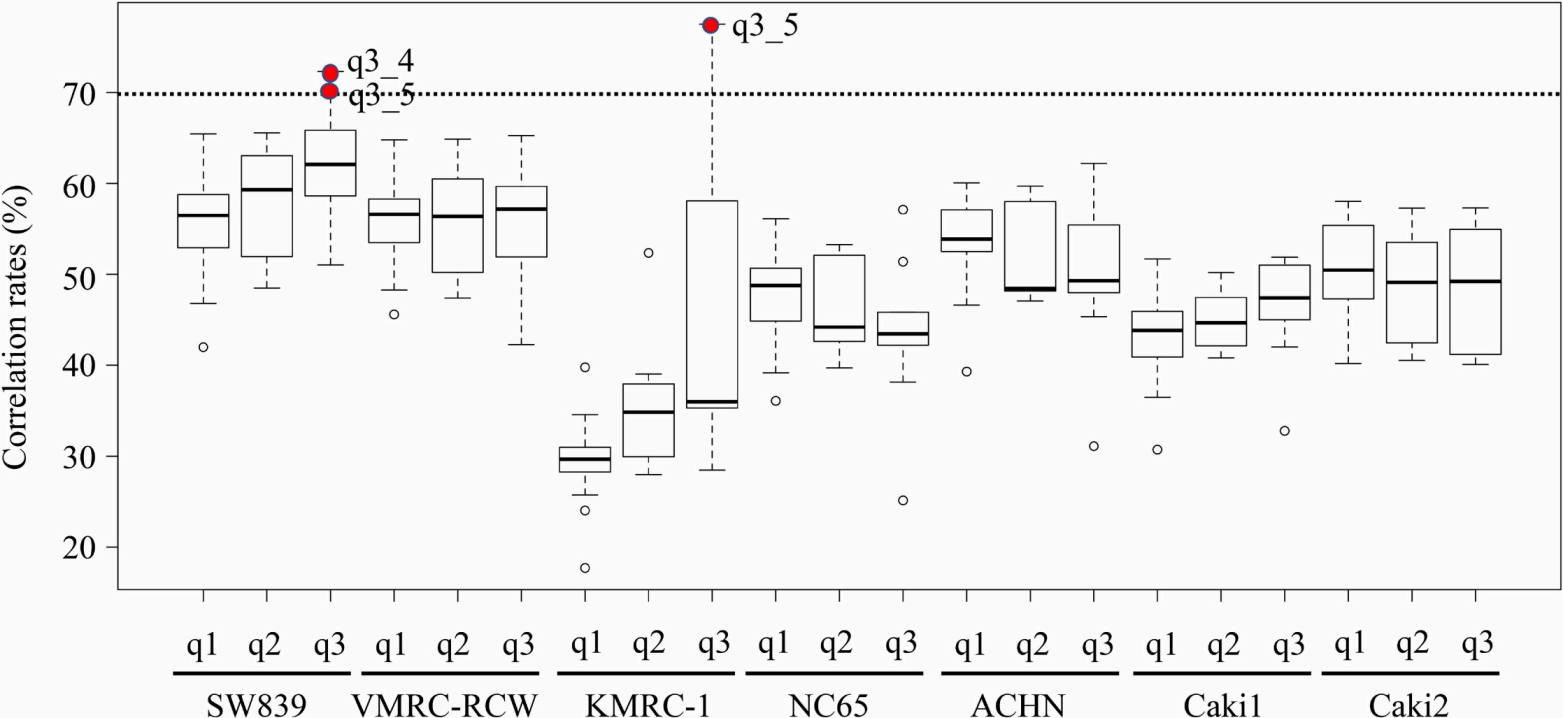
Correlation rates of global transcriptional expression in seven RCC cell lines with 43 tissues of RCC patients by genes count values using genes.count_table of Cuffnorm. In the box and whisper plot, solid lines and deviation bars indicated the mean correlation rates and variance. Red rods indicated tissue samples with a > 70% correlation rate for RCC cell lines. Group q1: 27 cases in disease-free status without metastases 5 years after initial surgery for resection of the primary RCC lesion. Group q2: seven cases with survival ≥ 4 years after initial diagnosis of metastasis. Group q3: nine cases with survival ≤ 2 years after initial diagnosis of metastasis.

Global transcriptional analysis was performed using seven RCC cell lines and 43 primary tissues of patients with RCC using previous data deposited in the DNA Data Bank of Japan (DDBJ) Japanese Genotype-phenotype Archive for genetic and phenotypic human data under accession number JGAS00000000149. This data contained 43 primary tissues and showed the following as previously reported [14]: 27 cases in disease-free status without metastases 5 years after initial surgery for resection of the primary RCC lesion (Group q1); seven cases with survival ≥ 4 years after initial diagnosis of metastasis (Group q2); and nine cases with survival ≤ 2 years after initial diagnosis of metastasis (Group q3, chemotherapy-resistant RCCs). The correlation rates of global transcriptional expression in seven RCC cell lines to 43 tissues of RCC patients were determined by genes count values using genes.count_table of Cuffnorm. SW839 showed a > 70% correlation rate for samples number four and five in Group q3 (q3_4 and 5) and KMRC-1 also showed a > 70% correlation rate for q3_5 (Fig 1). Three tissues of Group q3 (q3_4, 5 and 6) and one tissue of Group q2 (q2_3) were significantly correlated with starvation-resistant cell lines compared with starvation-sensitive cell lines (Table 1). The starvation-resistant cell lines, SW839 and KMRC-1, were significantly correlated with Group q3 vs q1. However, VMRC-RCW, the remaining starvation-resistant cell line, did not show a correlation with the four starvation-sensitive cell lines (Table 2). These results suggested that the two starvation-resistant RCC cell lines, SW839 and KMRC-1, were strongly correlated with and indicated a poor prognosis for patients with chemotherapy-resistant RCC (Group q3), which were resistant to treatment with tyrosine kinase and mTOR inhibitors [14].

**Table 1:** The average of correlation rates of global transcriptional expression in four tissues that showed significantly higher rates for Starvation-resistant RCC cell lines than starvation-sensitive RCC cell lines.

Tissue	Resistant	Sensitive	R/S	TTEST
**q2_3**	0.5808087	0.45290604	1.2824044	0.02200383
**q3_4**	0.62521217	0.465315	1.3436321	0.03198309
**q3_5**	0.63349868	0.32544917	1.94653647	0.02563367
**q3_6**	0.57662795	0.44876038	1.28493509	0.01260431

The average of correlation rates of three starvation-resistant and four -sensitive RCC cell lines are shown in the Resistant and Sensitive groups, respectively. R/S indicates the average of the correlation rates of starvation-resistant RCC cell lines per the average of the correlation rates of starvation-sensitive RCC cell lines.

**Table 2:** The average of correlation rates of global transcriptional expression in seven RCC cell lines to 43 tissues of RCC patients.

Cell line	q1	q2	q3
**Starvation-resistant**			
**SW839**	55.6 ± 1.0	57.6 ± 2.4	**62.2 ± 2.2**
**KMRC-1**	30.0 ± 0.8	35.8 ± 3.2	**45.2 ± 5.4**
**VMRC-RCW**	56.0 ± 0.9	55.7 ± 2.3	55.1 ± 2.4
**Starvation-sensitive**			
**NC65**	48.0 ± 0.9	46.7 ± 2.1	43.3 ± 2.9
**ACHN**	53.7 ± 0.9	52.5 ± 2.1	50.0 ± 0.9
**Caki1**	43.4 ± 0.9	45.0 ± 1.4	46.4 ± 2.0
**Caki2**	50.5 ± 1.0	48.4 ± 2.4	48.7 ± 2.4

Group q1: 27 cases in disease-free status without metastases 5 years after initial surgery for resection of the primary RCC lesion.

Group q2: seven cases with survival ≥ 4years after initial diagnosis of metastasis.

Group q3: nine cases with survival ≤ 2 years after initial diagnosis of metastasis.

Bold text indicates p < 0.05, pair-wise comparisons using *t*-tests with pooled SD vs q1 following ANOVA: SW839, F (2, 40) = 4.464, p = 1.779e^-2^; KMRC-1, F (2, 40) = 11.657, p = 1.026e^-4^.

### Principal component analysis of global transcriptomic expression in primary tissues of patients with RCC and RCC cell lines

**Fig 2:**
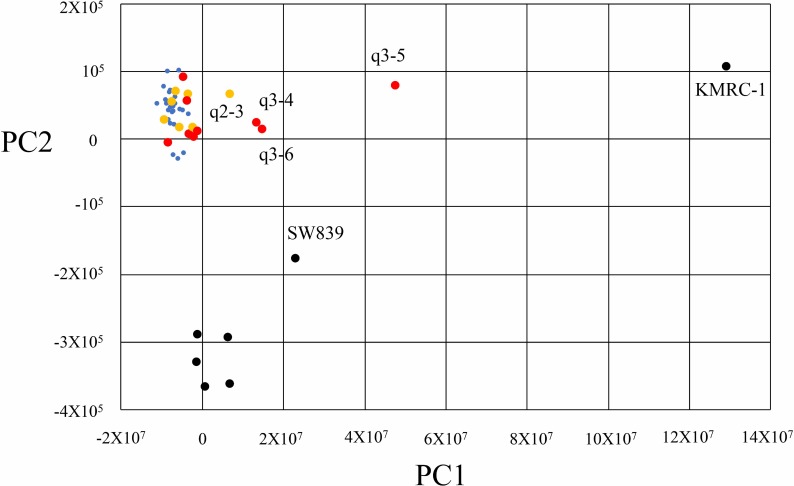
Principal component analysis of 43 tissues from RCC patients and seven RCC cell lines. The principal component analysis of 50 samples containing 43 primary tissues of patients with RCC and seven RCC cell lines was performed using the prcomp package of free software R (https://www.r-project.org/) using genes.count_table following Cuffnorm. Small blue, yellow, and red dots indicate Group q1, 27 cases in disease-free status without any metastasis 5 years after initial surgery for resection of a primary RCC lesion; Group q2, seven cases with survival ≥ 4 years after initial diagnosis of metastasis; and Group q3, nine cases with survival ≤ 2 years after initial diagnosis of metastasis [14], respectively. Black dots indicate cell lines. The two starvation-resistant RCC cell lines, SW839 and KMRC-1, were strongly correlated to q3_4, q3_5, and q3_6 compared with the other groups.

Principal component analysis of global transcriptional expression with 50 samples containing 43 primary tissues of patients and seven RCC cell lines was performed (Fig 2). Four tissues, q2_3, q3_4, q3_5, and q3_6, three starvation-resistant cell lines, SW839, KMRC-1, and VMRC-RCW, and two starvation-sensitive cell lines, Caki1and Caki2, showed a positive score on Principal component 1 (contribution rate: 39.7%). Four tissues, q2_3, q3_4, q3_5, and q3_6, and KMRC-1, a starvation-resistant cell line, showed a positive score on Principal component 2 (contribution rate: 12.7%). In both Principal component 1 and 2, the highest principal component loading gene was *FN1* (Table 3). These results suggested poor prognosis tissues of patients with RCC (Group q3) were separated into Subgroup q3A: q3_4, q3_5, and q3_6, whereas Subgroup q3B contained the other six samples. Furthermore, the two starvation-resistant RCC cell lines, SW839 and KMRC-1, were strongly correlated and emulated Subgroup q3A.

**Table 3:** The top 20 genes with highest loading in the principal component analysis of global transcriptional expression with seven RCC cell lines and 43 tissues from RCC patients.

PC1		PC2	
Gene name	Loading	Gene name	Loading
*FN1*	0.9124267	*FN1*	0.2542112
*SPARC*	0.0228946	*POLH*, *VEGFA*	0.2410031
*IGFBP3*	0.049493	*B2M*	0.2357109
*C3*	0.0111938	*CD74*	0.1520896
*TGFBI*	0.0828214	*RGS5*, *RP11-267N12*.*3*	0.1454602
*MT-ATP6 et al*.^a^	0.0450265	*NEAT1*	0.1133105
*COL1A1*	0.0057541	*SPARC*	0.1087097
*IGKC et al*.^b^	0.0098868	*GPX3*	0.0946695
*TNFSF10*	0.0076392	*IGFBP3*	0.0832647
*DCN*	0.0084225	*A2M*	0.0779432
*THBS1*	0.0276325	*SERPINA1*	0.0669899
*MIR4461 et al*.^c^	0.0056074	*IGFBP5*	0.0634394
*APOL1*	0.0141492	*DUSP1*, *RP11-779O18*.*2*	0.0575396
*TPM1*	0.0413184	*SNORA31*, *TPT1*	0.0566399
*MTATP6P1 et al*.^d^	0.0144129	*C3*	0.053693
*CLDN2*	0.0074486	*NDRG1*	0.052348
*MTND2P28*	0.011957	*TXNIP*	0.0517574
*AL121987*.*1*, *PEA15*	0.014757	*COL4A1*	0.0501314
*SLC34A2*	0.0088398	*TGFBI*	0.0434206
*AC079466*.*1*	0.0070826	*DDX17*	0.0431358

^a^TMT-ATP6, MT-ATP8, MT-CO1, MT-CO2, MT-CO3, MT-ND3, MT-ND4, MT-ND4L, MT-ND5, MT-TD, MT-TG, MT-TH, MT-TK, MT-TL2, MT-TR, MT-TS2.

^b^AC096579.13, AC096579.7, IGKC, IGKJ1, IGKJ2, IGKJ4, IGKJ5.

^c^CTB-36O1.3, MIR4461, MTND4P12.

^d^MTATP6P1, MTATP8P1, RP5-857K21.11.

### Global transcriptomic analysis in three chemotherapy-resistant RCC tissues and SW839 and KMRC-1 cell lines

Global transcriptional analysis was performed to profile Group A, three chemotherapy-resistant RCC tissues (Subgroup q3A) and SW839 and KMRC-1, relative to Group B containing the other 45 samples. We analyzed genes that were differentially expressed between Group A and B using Cuffdiff and identified 164 genes that were significantly up-regulated in Group A compared with Group B (S1 Table). These 164 genes were analyzed by KEGG pathway analysis and GO analysis using DAVID. KEGG pathway analysis showed that extracellular matrix (ECM)-related and cell cycle-related pathways were up-regulated in Group A (Table 4). *TGFB2*, *ITGB1*, *FN1* and *COL1A1* contributed to many pathways. GO analysis showed that mitosis- and cytokinesis-related genes were up-regulated in Group A (Table 5). These results suggested that three chemotherapy-resistant RCC tissues (Subgroup q3A) and two starvation-resistant RCC cell lines, SW839 and KMRC-1, showed a common malignant phenotype with invasive and proliferating activity.

**Table 4:** List of KEGG pathway analysis results for up-regulated genes in Group A^a^ compared with Group B^b^.

Term	p value	Gene name
ECM-receptor interaction	0.00000	*LAMB3*, *CD44*, *LAMC2*, *ITGA3*, *COL1A1*, *LAMC1*, *ITGB1*, *COL11A1*, *COL5A1*, *HMMR*, *FN1*
Cell cycle	0.00001	*CDC6*, *CDC45*, *BUB1*, *BUB1B*, *TTK*, *CHEK1*, *ORC6*, *CDC27*, *MCM4*, *TGFB2*
Amoebiasis	0.00003	*LAMB3*, *IL6*, *LAMC2*, *COL1A1*, *LAMC1*, *COL11A1*, *COL5A1*, *TGFB2*, *FN1*
Focal adhesion	0.00072	*PAK6*, *LAMB3*, *LAMC2*, *ITGA3*, *COL1A1*, *LAMC1*, *ITGB1*, *COL11A1*, *COL5A1*, *FN1*
PI3K-Akt signaling pathway	0.00200	*LAMB3*, *IL6*, *CREB3L1*, *LAMC2*, *ITGA3*, *COL1A1*, *LAMC1*, *ITGB1*, *COL11A1*, *BRCA1*, *COL5A1*, *FN1*
Hypertrophic cardiomyopathy	0.00281	*IL6*, *ITGA3*, *TPM1*, *ITGB1*, *TPM4*, *TGFB2*
Small cell lung cancer	0.00418	*LAMB3*, *LAMC2*, *ITGA3*, *LAMC1*, *ITGB1*, *FN1*
Prion diseases	0.00661	*NCAM1*, *IL6*, *LAMC1*, *HSPA5*
Toxoplasmosis	0.01361	*LAMB3*, *LAMC2*, *LAMC1*, *ITGB1*, *HSPA8*, *TGFB2*
Dilated cardiomyopathy	0.01958	*ITGA3*, *TPM1*, *ITGB1*, *TPM4*, *TGFB2*
Hippo signaling pathway	0.03355	*WNT5A*, *PARD3*, *FRMD6*, *TP53BP2*, *SERPINE1*, *TGFB2*
Thyroid hormone synthesis	0.04539	*CREB3L1*, *GPX8*, *HSPA5*, *TTF2*

^a^Three poor prognosis tissues (q3_4, 5, 6) and SW839 and KMRC-1.

^b^The other 45 samples.

**Table 5:** List of GO analysis results for up-regulated genes in Group A^a^ compared with Group B^b^.

Term	p value	Gene name
DNA replication initiation	0.00000	*CDC6*, *CDC45*, *ORC6*, *POLA2*, *MCM10*, *MCM4*
Chromosome segregation	0.00008	*HJURP*, *NEK2*, *SKA3*, *SKA2*, *BRCA1*, *ESCO2*
Protein localization to kinetochore	0.00008	*BUB1B*, *SPDL1*, *AURKB*
Positive regulation of cytokinesis	0.00020	*KIF23*, *CDC6*, *AURKB*, *ECT2*
Spindle checkpoint	0.00031	*SPDL1*, *AURKB*
Cell division	0.00056	*CDC6*, *CDC45*, *NCAPH*, *SPDL1*, *SKA2*, *CDC27*
Mitotic spindle midzone assembly	0.00210	*KIF23*, *AURKB*
Collagen fibril organization	0.00283	*CYP1B1*, *COL1A1*, *COL11A1*, *TGFB2*
DNA replication	0.00307	*MCM8*, *ORC6*, *POLA2*, *MCM10*, *BRCA1*
Regulation of mitotic metaphase/ anaphase transition	0.00307	*MCM8*, *ORC6*, *POLA2*, *MCM10*, *BRCA1*
Mitotic chromosome condensation	0.00442	*NCAPH*, *NUSAP1*, *SMC4*
Positive regulation of telomere capping	0.00751	*NEK2*, *AURKB*
Positive regulation of angiogenesis	0.00905	*WNT5A*, *CYP1B1*, *F3*, *SERPINE1*, *BRCA1*

^a^Three poor prognosis tissues (q3_4, 5, 6) and SW839 and KMRC-1.

^b^The other 45 samples.

### Higher fibronectin (*FN1*) expression predicts a poor prognosis in patients with chemotherapy-resistant RCC

**Fig 3:**
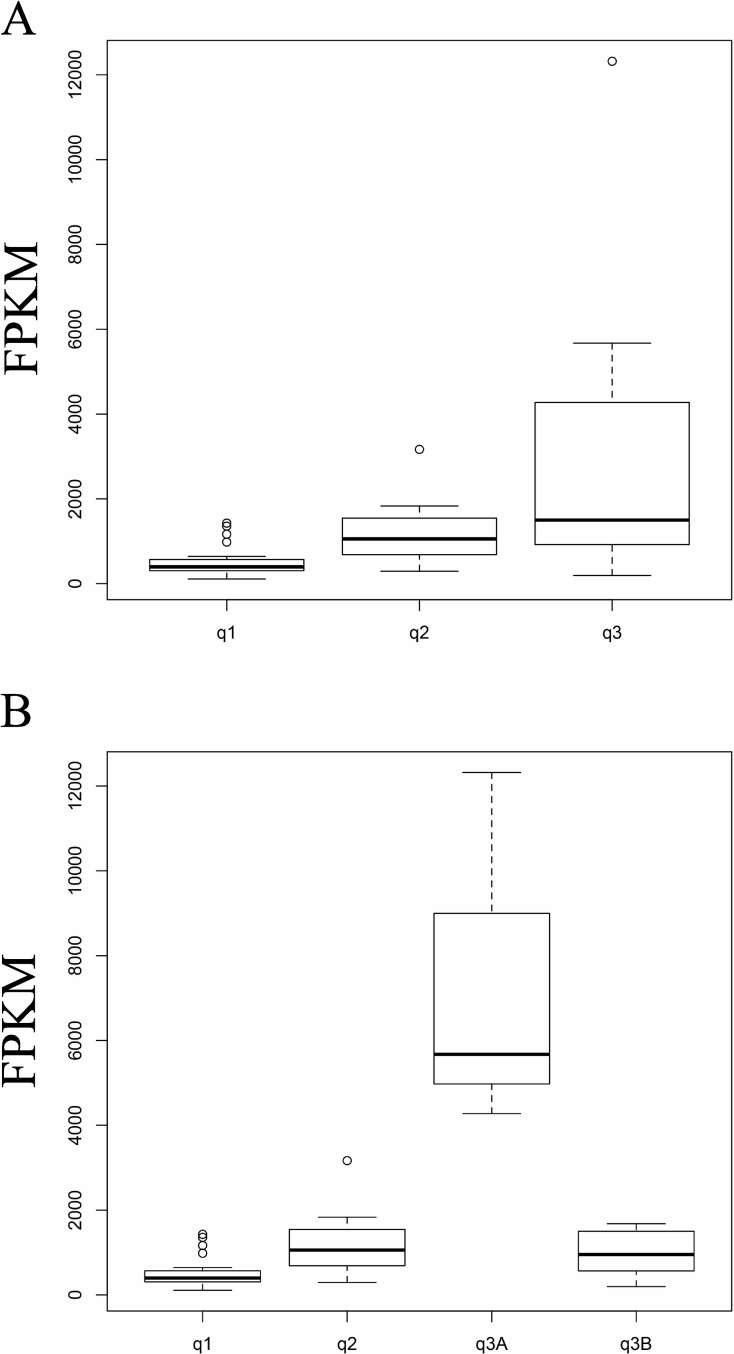
Transcriptional comparison of *FN1* with FPKM values by global transcriptomic analyses of primary tissues obtained from patients with RCC. In the box and whisper plot, solid lines and deviation bars indicated the mean FPKM and variance. (A) The box and whisper plots of three groups. FPKM values of *FN1* were determined in 43 primary tissues with RCC and the cases were categorized into three Groups: Group q1, 27 cases in disease-free status without any metastasis 5 years after initial surgery for resection of a primary RCC lesion; Group q2, seven cases with survival ≥ 4 years after initial diagnosis of metastasis; and Group q3, nine cases with survival ≤ 2 years after initial diagnosis of metastasis [14]. ANOVA: F (2, 40) = 7.1437, p = 0.002224; pair-wise comparisons using *t*-tests with pooled SD showed that Group q3 had significantly higher *FN1* expression than cases in Groups q1 (p = 0.0015). (B) The box and whisper plots of four groups. Group q3 was separated into Subgroup q3A: q3_4, q3_5, and q3_6, whereas Subgroup q3B contained the other six samples. ANOVA: F (3, 39) = 35.112, p = 3.624e^-11^; pair-wise comparisons using *t*-tests with pooled SD showed that Group q3A had significantly higher *FN1* expression than cases in Groups q1, q2 and q3B (p = 7.5e^-12^, 4.3e^-9^, 3.0e^-9^, respectively).

**Fig 4:**
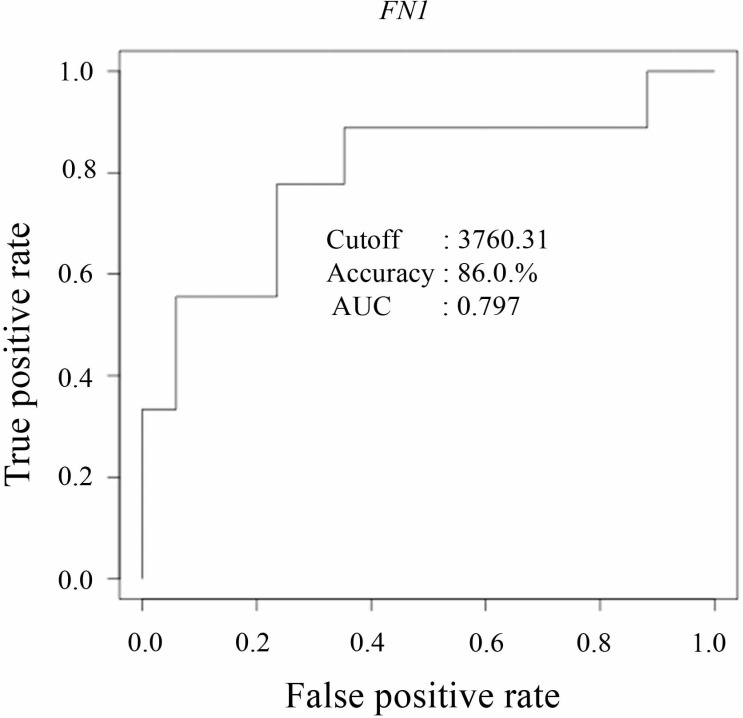
Receiver Operating Characteristic (ROC) curve analysis of the FPKM transcriptional value of *FN1* in 43 primary tissues obtained from patients with RCC. ROC analysis revealed nine cases with poor prognosis (i.e., survival ≤ 2 years after initial diagnosis of metastasis) versus 34 cases with good prognosis (i.e., survival ≥ 5 years without metastases or survival ≥ 4 years after initial diagnosis of metastasis). *FN1* differentiated between cases with poor and good prognosis with an accuracy of 86.0%.

*FN1* was the highest principal component loading gene (Table 3) and contained many up-regulated pathways in Group A (Table 4). Therefore, the present study evaluated the predictive value of *FN1* for poor prognosis in 43 primary tissues from patients with RCC and seven RCC cell lines. RCC samples obtained from Group q3 (i.e., survival ≤ 2 years after initial diagnosis of metastasis) had significantly higher *FN1* expression compared with samples in Group q1 surviving ≥ 5 years without metastases (Fig 3A). In addition, Subgroup q3A had significantly higher *FN1* expression compared with Groups q1, q2, and Subgroup q3B (Fig 3B). Moreover, ROC curve analysis of 43 patients in this cohort (Fig 4 and Table 6) showed that *FN1* predicted patient mortality caused by RCC within 2 years after the initial diagnosis of metastasis with a high maximum accuracy of 86.0% based on the cut-off FPKM value (minimum value of FPKM that showed maximum accuracy) of 3760.31. This cut-off FPKM value for poor prognosis (Group q3, survival ≤ 2 years after initial diagnosis of metastasis) separated Subgroup q3A from the other groups. Therefore, the maximum accuracy of predicting patient mortality in 16 patients with metastasis was low (0.625). However, *FN1* predicted the development of metastasis in 43 patients with a high maximum accuracy of 0.814 based on the lower cut-off FPKM value of 750.53. These results showed that *FN1* might be a useful predictive biomarker for poor prognosis and the development of metastasis in patients with RCC. However, *FN1* might be a specific index marker of Subgroup q3A, which formed part of the poor prognosis patient group with chemotherapy-resistant RCC.

**Table 6:** List of the receiver operating characteristic (ROC) curve analysis, which predicted poor prognosis in RCC with maximum accuracy, using FPKM transcriptional values of *FN1*.

	Cutoff	TP	FP	FN	TN	Sensitivity	Specificity	Accuracy	AUC
**q2+q3 vs q1**	750.53	12	4	4	23	0.75	0.852	0.814	0.829
**q3 vs q1+q2**	3760.31	3	0	6	34	0.333	1.000	0.860	0.797
**q3 vs q2**	3760.31	3	0	6	7	0.333	1.000	0.625	0.619
**q3A vs q3B**	3760.31	3	0	0	6	1.000	1.000	1.000	1.000

ROC analysis was performed using 43 primary RCC tissues containing nine patients with poor prognosis (i.e., survival ≤ 2 years after initial diagnosis of metastasis) and 34 patients with good prognosis (i.e., survival ≥ 5 years without or ≥ 4 years with metastases). The FPKM transcriptional values of *FN1* were significantly higher in the left group versus the right group.

Cutoff: minimum value of FPKM that showed maximum accuracy, TP: true positive, FP: false positive, FN: false negative, TN: true negative, AUC: area under the curve

## Discussion

This study showed that starvation-resistant RCC cell lines, SW839 and KMRC-1, were strongly correlated and emulated primary tissues from poor prognosis patients with chemotherapy-resistant RCC, and indicated their characteristics of chemotherapy-resistant RCC, which was useful for the search of markers to predict poor prognosis and the development of therapeutic agents and their index markers for chemotherapy-resistant RCCs.

The global transcriptional analysis of 43 primary tissues from patients with RCC and seven RCC cell lines showed that starvation-resistant RCC cell lines, SW839 and KMRC-1, were strongly correlated to primary tissues of poor prognosis patients with chemotherapy-resistant RCC, especially three of nine primary tissues from RCC patients. Their cell lines and tissues formed a group (Group A) that had a common malignant phenotype of maintaining invasive and proliferating activities compared with other cell lines and tissues as determined by their gene expression pattern. We previously reported that starvation-sensitive RCC cell lines showed G2/M-phase arrest under glucose deprivation leading to cell death, but that starvation-resistant RCC cell lines containing SW839 and KMRC-1 survived under glucose deprivation without the induction of G2/M transition-arrest [10]. This report is consistent with the result that mitosis-related genes were up-regulated in Group A. Therefore, chemotherapy-resistant RCCs may escape cell death induced by G2/M transition-arrest in the tumor microenvironment where nutrients such as glucose are deficient. Another previous study reporting that KMRC-1was highly invasive [14] was also consistent with the result whereby ECM-related genes were up-regulated in Group A. These results suggest that the characteristics of SW839 and KMRC-1 strongly emulated chemotherapy-resistant RCCs.

SW839, KMRC-1, and primary tissues from poor prognosis patients with chemotherapy-resistant RCC up-regulated expression of *FN1*, an ECM-related gene, compared with other cell lines and tissues. Therefore, this study indicates that *FN1* expression might be a useful marker to predict a poor prognosis as previously reported [19]. However, our results showed that *FN1* expression was a predictive biomarker of a group of poor prognosis patients with chemotherapy-resistant RCC, termed Subgroup q3A. Makers for specific subgroup of patients will be useful in combination with markers used to cover total patients. For patients with RCC, *FN1* expression will be useful in combination with a predictive biomarker of poor prognosis such as *ARL4C*, which predicts patient mortality caused by RCC within 2 years after the initial diagnosis of metastasis with a high accuracy of 97.7% [14]. SW839 and KMRC-1 were useful in the search for a marker to predict a poor prognosis in patients with RCC. Some genes specifically up-regulated in SW839 and KMRC-1 were also candidates for poor prognosis markers in chemotherapy-resistant RCCs.

Starvation-resistant RCC cell lines, including VMRC-RCW as well as SW839 and KMRC-1, were previously reported to undergo cell death induced by buformin (a biguanide) [10, 11], etomoxir (an inhibitor of beta-oxidation from fatty acids) [11], and chetomin (a nuclear inhibitor of hypoxia inducible factor [HIF]) [13]. These drugs may be potential therapeutic agents for malignant RCCs. Therefore, drug screening using starvation-resistant RCC cell lines will be useful for the development of therapeutic agents for malignant RCCs. Of note, drug repositioning (e.g. biguanide) using SW839 and KMRC-1 may be effective.

In the last few decades, the treatment for advanced RCC has evolved dramatically due to the introduction of targeted therapies and novel immunotherapies using checkpoint inhibitors. The CheckMate-214 trial showed the survival superiority of combined immunotherapy over targeted therapy in a cohort of patients at intermediate or poor-risk [20]. *CD274* (PDL1), a target of checkpoint inhibitors, was an up-regulated gene in Group A compared with Group B (S1 Table). Therefore, SW839 and KMRC-1 may be effective for the search of checkpoint inhibitors.

It is important to identify index markers for the clinical application of potential therapeutic agents. The connection of data from global transcriptional analysis of clinical RCC tissues and RCC cell lines will identify candidate index markers of therapeutic agents, which can be screened using the KMRC-1 and SW839 cell lines.

## Conclusions

This study showed that starvation-resistant RCC cell lines, SW839 and KMRC-1, emulated chemotherapy-resistant RCC and therefore will be useful for identifying markers to predict poor prognosis and the development of therapeutic agents and their index markers for chemotherapy-resistant RCCs.

## Supporting information

S1 Table List of genes that were significantly up-regulated in Group A^a^ compared with Group B^b^. ^a^Three poor prognosis tissues (q3_4, 5, 6) and SW839 and KMRC-1. ^b^The other 45 samples.
(DOCX)
