# Supplementary material for: Analysis of the characteristics of chemotherapy-resistant renal cell carcinomas based on global transcriptional analysis of their tissues and cell lines
Source: PLoS One. 2019 Nov 27;14(11):e0225721. doi: 10.1371/journal.pone.0225721 (PMC6881023; doi:10.1371/journal.pone.0225721)
Supplement: S1 Table — aThree poor prognosis tissues (q3_4, 5, 6) and SW839 and KMRC-1. bThe other 45 samples. (DOCX) [file pone.0225721.s001.docx]

**Supporting information**

**S1 Table.** **List of genes which were significantly up-regulated in Group A^a^ compared with Group B^b^**.

|  | **Average of RPKM value** | |  |  |  |
| --- | --- | --- | --- | --- | --- |
| **Gene name** | **Group A** | **Group B** | **Fold_change (log2)** | **p_value** | **q_value** |
| *CDK15* | 0.319 | 13.115 | 5.363 | 0.000 | 0.006 |
| *COL11A1* | 2.094 | 73.762 | 5.139 | 0.000 | 0.006 |
| *UCA1* | 0.979 | 30.578 | 4.966 | 0.000 | 0.006 |
| *LAMB3* | 6.521 | 170.957 | 4.712 | 0.000 | 0.006 |
| *ZNF365* | 1.384 | 26.539 | 4.262 | 0.000 | 0.006 |
| *ADAM12* | 2.409 | 36.712 | 3.930 | 0.000 | 0.006 |
| *F3* | 15.031 | 196.847 | 3.711 | 0.000 | 0.006 |
| *WNT5A* | 2.716 | 34.733 | 3.677 | 0.000 | 0.006 |
| *ANLN* | 23.887 | 299.825 | 3.650 | 0.000 | 0.006 |
| *FN1* | 602.536 | 7506.890 | 3.639 | 0.000 | 0.006 |
| *TGFB2* | 7.479 | 92.218 | 3.624 | 0.000 | 0.006 |
| *STAC* | 2.673 | 30.711 | 3.522 | 0.000 | 0.006 |
| *LAMC2* | 7.227 | 74.615 | 3.368 | 0.000 | 0.006 |
| *GFRA1* | 8.526 | 84.740 | 3.313 | 0.000 | 0.006 |
| *CKAP2L* | 3.074 | 29.651 | 3.270 | 0.000 | 0.006 |
| *CADM1* | 14.642 | 125.591 | 3.101 | 0.000 | 0.006 |
| *NCAM1* | 5.243 | 42.428 | 3.017 | 0.000 | 0.006 |
| *CLEC4E* | 19.262 | 145.796 | 2.920 | 0.000 | 0.006 |
| *CD274* | 4.549 | 34.013 | 2.903 | 0.000 | 0.006 |
| *TTK* | 3.913 | 27.852 | 2.831 | 0.000 | 0.006 |
| *CCDC80* | 49.642 | 334.115 | 2.751 | 0.000 | 0.006 |
| *UBE2C* | 27.786 | 183.929 | 2.727 | 0.000 | 0.006 |
| *CENPE* | 2.239 | 14.732 | 2.718 | 0.000 | 0.006 |
| *COL5A1* | 21.419 | 135.575 | 2.662 | 0.000 | 0.006 |
| *DEPDC5* | 20.025 | 124.902 | 2.641 | 0.000 | 0.006 |
| *HJURP* | 12.448 | 76.817 | 2.626 | 0.000 | 0.006 |
| *IGF2BP2* | 11.338 | 68.555 | 2.596 | 0.000 | 0.006 |
| *HMMR* | 8.282 | 49.467 | 2.578 | 0.000 | 0.006 |
| *IL6* | 28.797 | 170.836 | 2.569 | 0.000 | 0.006 |
| *CENPI* | 2.038 | 11.886 | 2.544 | 0.000 | 0.006 |
| *KIF6* | 0.635 | 3.548 | 2.483 | 0.000 | 0.006 |
| *TP53BP2* | 30.956 | 167.668 | 2.437 | 0.000 | 0.006 |
| *DEPDC1* | 4.872 | 25.715 | 2.400 | 0.000 | 0.006 |
| *LINC00511* | 4.227 | 22.193 | 2.392 | 0.000 | 0.006 |
| *BUB1* | 9.888 | 51.206 | 2.373 | 0.000 | 0.006 |
| *AURKA* | 10.966 | 56.249 | 2.359 | 0.000 | 0.006 |
| *GALNT7* | 15.589 | 78.593 | 2.334 | 0.000 | 0.006 |
| *COL1A1* | 89.404 | 450.260 | 2.332 | 0.000 | 0.006 |
| *MICAL2* | 27.256 | 126.108 | 2.210 | 0.000 | 0.006 |
| *PLK4* | 5.483 | 24.800 | 2.177 | 0.000 | 0.006 |
| *NCAPH* | 6.819 | 30.679 | 2.170 | 0.000 | 0.006 |
| *DCBLD2* | 67.897 | 298.010 | 2.134 | 0.000 | 0.006 |
| *ECT2* | 17.585 | 75.053 | 2.094 | 0.000 | 0.006 |
| *LAMC1* | 81.835 | 347.108 | 2.085 | 0.000 | 0.006 |
| *CD44* | 199.643 | 833.067 | 2.061 | 0.000 | 0.006 |
| *ENAH* | 36.594 | 148.814 | 2.024 | 0.000 | 0.006 |
| *ENTPD4, LOXL2* | 77.670 | 313.973 | 2.015 | 0.000 | 0.006 |
| *SMC4* | 47.542 | 189.172 | 1.992 | 0.000 | 0.006 |
| *ITGB1* | 391.835 | 1549.430 | 1.983 | 0.000 | 0.006 |
| *PARPBP* | 3.932 | 15.375 | 1.967 | 0.000 | 0.006 |
| *BUB1B, PAK6, RP11-133K1.2* | 11.525 | 44.758 | 1.957 | 0.000 | 0.006 |
| *PARD3* | 33.453 | 128.857 | 1.946 | 0.000 | 0.006 |
| *MLPH* | 12.750 | 48.779 | 1.936 | 0.000 | 0.006 |
| *PRC1* | 45.070 | 172.288 | 1.935 | 0.000 | 0.006 |
| *ORC6* | 6.889 | 25.314 | 1.877 | 0.000 | 0.006 |
| *OXCT1* | 36.669 | 133.729 | 1.867 | 0.000 | 0.006 |
| *CPD* | 52.116 | 188.664 | 1.856 | 0.000 | 0.006 |
| *CEP70* | 17.365 | 61.519 | 1.825 | 0.000 | 0.006 |
| *TPM4* | 363.701 | 1238.660 | 1.768 | 0.000 | 0.006 |
| *ITGA3* | 150.477 | 501.149 | 1.736 | 0.000 | 0.006 |
| *RAD51AP1* | 9.058 | 28.299 | 1.643 | 0.000 | 0.006 |
| *NFKBIZ, NXPE3* | 50.281 | 153.408 | 1.609 | 0.000 | 0.006 |
| *BRCA1* | 7.012 | 20.634 | 1.557 | 0.000 | 0.006 |
| *PSMD2* | 150.409 | 442.466 | 1.557 | 0.000 | 0.006 |
| *NPC1* | 54.110 | 155.659 | 1.524 | 0.000 | 0.006 |
| *CDC42EP2, POLA2* | 15.684 | 44.244 | 1.496 | 0.000 | 0.006 |
| *CHEK1, STT3A* | 134.334 | 375.310 | 1.482 | 0.000 | 0.006 |
| *APCDD1L-AS1* | 0.916 | 13.855 | 3.918 | 0.000 | 0.010 |
| *TENM3* | 3.461 | 31.038 | 3.165 | 0.000 | 0.010 |
| *OSBPL6* | 0.982 | 6.325 | 2.687 | 0.000 | 0.010 |
| *SCG5* | 8.093 | 46.914 | 2.535 | 0.000 | 0.010 |
| *NEK2* | 6.690 | 30.733 | 2.200 | 0.000 | 0.010 |
| *ZWINT* | 19.139 | 72.542 | 1.922 | 0.000 | 0.010 |
| *MELK* | 8.445 | 30.694 | 1.862 | 0.000 | 0.010 |
| *FRMD6* | 12.118 | 37.360 | 1.624 | 0.000 | 0.010 |
| *R3HDM1* | 26.706 | 77.986 | 1.546 | 0.000 | 0.010 |
| *ARHGAP11A* | 9.489 | 26.360 | 1.474 | 0.000 | 0.010 |
| *HDLBP* | 463.031 | 1128.930 | 1.286 | 0.000 | 0.010 |
| *TPM1* | 623.919 | 1477.030 | 1.243 | 0.000 | 0.010 |
| *ABCB5* | 0.670 | 17.890 | 4.738 | 0.000 | 0.014 |
| *-* | 0.412 | 4.147 | 3.330 | 0.000 | 0.014 |
| *HSF2BP* | 0.758 | 5.265 | 2.797 | 0.000 | 0.014 |
| *CENPF* | 10.032 | 67.154 | 2.743 | 0.000 | 0.014 |
| *S100A2* | 10.587 | 62.949 | 2.572 | 0.000 | 0.014 |
| *ADAMTS6* | 1.322 | 5.496 | 2.056 | 0.000 | 0.014 |
| *DTL* | 6.357 | 24.909 | 1.970 | 0.000 | 0.014 |
| *TGFBI* | 748.034 | 2661.470 | 1.831 | 0.000 | 0.014 |
| *GPX8* | 35.212 | 124.090 | 1.817 | 0.000 | 0.014 |
| *MBOAT2* | 14.180 | 42.720 | 1.591 | 0.000 | 0.014 |
| *NUSAP1* | 40.485 | 114.347 | 1.498 | 0.000 | 0.014 |
| *MCM4* | 94.154 | 264.509 | 1.490 | 0.000 | 0.014 |
| *LINC00607* | 0.478 | 2.870 | 2.585 | 0.000 | 0.017 |
| *CREB3L1* | 2.517 | 12.487 | 2.311 | 0.000 | 0.017 |
| *SGOL1* | 3.150 | 11.195 | 1.829 | 0.000 | 0.017 |
| *RBFOX3* | 0.316 | 1.100 | 1.798 | 0.000 | 0.017 |
| *ITGB6* | 15.506 | 52.717 | 1.765 | 0.000 | 0.017 |
| *ACACA* | 19.391 | 43.550 | 1.167 | 0.000 | 0.017 |
| *HS3ST3A1* | 0.677 | 7.067 | 3.383 | 0.000 | 0.020 |
| *AC108142.1* | 1.041 | 9.673 | 3.216 | 0.000 | 0.020 |
| *MEGF11* | 3.794 | 27.216 | 2.843 | 0.000 | 0.020 |
| *CYP1B1* | 15.007 | 91.173 | 2.603 | 0.000 | 0.020 |
| *PROM2* | 1.520 | 8.215 | 2.434 | 0.000 | 0.020 |
| *KIF23* | 9.386 | 42.164 | 2.167 | 0.000 | 0.020 |
| *DIAPH3* | 5.013 | 20.374 | 2.023 | 0.000 | 0.020 |
| *CENPU* | 12.146 | 44.880 | 1.886 | 0.000 | 0.020 |
| *CTD-2510F5.6, PRR11, SMG8* | 28.257 | 85.458 | 1.597 | 0.000 | 0.020 |
| *CENPL* | 4.938 | 14.704 | 1.574 | 0.000 | 0.020 |
| *SSR3* | 141.367 | 400.978 | 1.504 | 0.000 | 0.020 |
| *HSPA8* | 641.313 | 1700.070 | 1.407 | 0.000 | 0.020 |
| *KPNA2* | 60.528 | 263.156 | 2.120 | 0.000 | 0.022 |
| *C1orf204, CCDC19, RP11-190A12.7, VSIG8* | 8.272 | 32.673 | 1.982 | 0.000 | 0.022 |
| *CALU* | 119.785 | 466.839 | 1.962 | 0.000 | 0.022 |
| *SPDL1* | 18.479 | 63.549 | 1.782 | 0.000 | 0.022 |
| *HSPA5* | 123.808 | 399.273 | 1.689 | 0.000 | 0.022 |
| *PSD3* | 3.639 | 11.111 | 1.611 | 0.000 | 0.022 |
| *GULP1* | 33.820 | 99.070 | 1.551 | 0.000 | 0.022 |
| *AURKB* | 13.809 | 34.366 | 1.315 | 0.000 | 0.022 |
| *KIFC1* | 7.799 | 44.403 | 2.509 | 0.000 | 0.024 |
| *RRM2* | 23.251 | 111.439 | 2.261 | 0.000 | 0.024 |
| *CDC45* | 6.236 | 25.317 | 2.022 | 0.000 | 0.024 |
| *CPNE7* | 6.646 | 26.488 | 1.995 | 0.000 | 0.024 |
| *AMACR, C1QTNF3, RP11-1084J3.4* | 61.280 | 183.514 | 1.582 | 0.000 | 0.024 |
| *MCM10* | 2.325 | 14.636 | 2.654 | 0.000 | 0.026 |
| *C3orf55* | 3.235 | 19.909 | 2.621 | 0.000 | 0.026 |
| *GPNMB* | 174.159 | 760.166 | 2.126 | 0.000 | 0.028 |
| *FADS1* | 25.736 | 110.470 | 2.102 | 0.000 | 0.028 |
| *GPM6A* | 3.661 | 17.972 | 2.295 | 0.001 | 0.030 |
| *RUNX2* | 6.008 | 23.580 | 1.973 | 0.001 | 0.030 |
| *PLOD2* | 162.796 | 517.553 | 1.669 | 0.001 | 0.030 |
| *FADS2, FEN1* | 31.876 | 99.014 | 1.635 | 0.001 | 0.030 |
| *CHRDL1* | 3.159 | 49.421 | 3.968 | 0.001 | 0.032 |
| *RP11-150O12.1, RP11-150O12.6, RP11-527N22.2* | 1.097 | 8.804 | 3.005 | 0.001 | 0.032 |
| *DNMT1, S1PR2* | 63.500 | 190.927 | 1.588 | 0.001 | 0.032 |
| *WHSC1* | 21.446 | 60.497 | 1.496 | 0.001 | 0.032 |
| *EPB41L2* | 77.991 | 180.967 | 1.214 | 0.001 | 0.032 |
| *ZNF474* | 0.427 | 2.738 | 2.681 | 0.001 | 0.034 |
| *CDC6* | 7.856 | 33.396 | 2.088 | 0.001 | 0.034 |
| *HOMER2* | 4.197 | 32.027 | 2.932 | 0.001 | 0.036 |
| *SKA2* | 44.101 | 158.295 | 1.844 | 0.001 | 0.036 |
| *CDC27* | 45.234 | 131.769 | 1.543 | 0.001 | 0.036 |
| *TTF2* | 14.559 | 35.721 | 1.295 | 0.001 | 0.036 |
| *EPHB1* | 1.309 | 7.234 | 2.466 | 0.001 | 0.039 |
| *SKA3* | 4.429 | 31.875 | 2.847 | 0.001 | 0.041 |
| *SGOL2* | 2.992 | 16.017 | 2.420 | 0.001 | 0.041 |
| *PEG10* | 27.375 | 140.731 | 2.362 | 0.001 | 0.041 |
| *HNRNPR* | 68.438 | 181.881 | 1.410 | 0.001 | 0.041 |
| *SH3GL3* | 0.187 | 1.511 | 3.011 | 0.001 | 0.042 |
| *TFPI2* | 48.416 | 373.012 | 2.946 | 0.001 | 0.042 |
| *CALR* | 503.066 | 1362.270 | 1.437 | 0.001 | 0.042 |
| *PCDH9* | 0.211 | 2.011 | 3.255 | 0.001 | 0.044 |
| *NEIL3* | 2.871 | 15.454 | 2.428 | 0.001 | 0.044 |
| *ESCO2* | 2.406 | 12.781 | 2.409 | 0.001 | 0.044 |
| *P2RY6* | 3.334 | 10.784 | 1.694 | 0.001 | 0.044 |
| *SERPINE1* | 83.455 | 518.890 | 2.636 | 0.001 | 0.046 |
| *BDH1* | 5.568 | 16.207 | 1.541 | 0.001 | 0.046 |
| *TOP2A* | 24.510 | 142.940 | 2.544 | 0.001 | 0.047 |
| *ZNF385D* | 3.558 | 14.168 | 1.994 | 0.001 | 0.047 |
| *XRCC5* | 173.447 | 461.388 | 1.411 | 0.001 | 0.047 |
| *CAP1* | 217.941 | 506.033 | 1.215 | 0.001 | 0.047 |
| *ABI3BP* | 73.637 | 163.607 | 1.152 | 0.001 | 0.047 |
| *CACNA1G* | 0.090 | 0.591 | 2.713 | 0.001 | 0.048 |
| *PBK* | 7.796 | 50.704 | 2.701 | 0.001 | 0.048 |
| *MCM8* | 4.780 | 16.542 | 1.791 | 0.001 | 0.048 |

^a^Three poor prognosis tissues (q3_4, 5, 6) and SW839 and KMRC-1.

^b^The other 45 samples.
